# 5G Cloud‐Enabled Telemedicine for Remote Management of Heart Failure Patients With Implantable Cardioverter‐Defibrillators: A Multicenter Open‐Label Randomized Controlled Trial

**DOI:** 10.1002/mco2.71037

**Published:** 2026-09-29

**Authors:** Pan Li, Fayuan Fu, Yiding Qi, Xianxian Zhao, Yingyi Qin, Xinghua Shan, Jing Jiang, Guowei Wang, Jingjuan Huang, Bo Liu, Zhifu Guo, Zhongkai Wang

**Affiliations:** ^1^ Department of Cardiology Changhai Hospital Naval Medical University Shanghai China; ^2^ Department of Cardiology Union Hospital Fujian Medical University Fuzhou China; ^3^ Department of Cardiology Shanghai Chest Hospital Shanghai Jiao Tong University School of Medicine Shanghai China; ^4^ Department of Health Statistics Naval Medical University Shanghai China; ^5^ Department of Cardiology PLA Navy No. 971 Hospital Qingdao China; ^6^ Department of Cardiology Xinjiang Military Region General Hospital Urumqi China; ^7^ Department of Cardiology Xinhua Hospital Shanghai Jiao Tong University School of Medicine Shanghai China

**Keywords:** 5G cloud technology, cardiac resynchronization therapy defibrillator, implantable cardioverter defibrillator, heart failure

## Abstract

Postoperative follow‐up for patients with implantable cardioverter‐defibrillators and cardiac resynchronization therapy defibrillators (ICD/CRT‐D) is often delayed in remote areas because of geographic barriers and limited healthcare resources, increasing the risk of adverse outcomes. The “Make Changes” study, was a prospective, multicenter, open‐label randomized trial evaluating a 5G‐cloud technology platform (5G‐CTP) for real‐time remote device interrogation and reprogramming versus standard In‐clinic care. Between October 2022 and February 2024, 206 patients from five centers were randomly assigned to 12 months of follow‐up with 5G‐CTP (*n* = 104) or In‐clinic care (*n* = 102). For the primary endpoint of time from clinical event to intervention, the 5G‐CTP group had a significantly shorter response time than the In‐Clinic group (4.2 vs. 11.5 days; p < 0.001). For secondary endpoints, the 5G‐CTP group showed greater improvement in life quality at 12 months based on Minnesota Living with Heart Failure Questionnaire (*p* = 0.012). Rates of unplanned visits and clinical interventions were similar between groups, with no procedure‐related complications. This model integrates community hospitals with implantation centers through 5G networks, enabling timely specialist‐directed care locally without long‐distance travel. It is a feasible strategy for postoperative ICD/CRT‐D management, particularly in underserved regions with limited access to specialized cardiac care.

## Graphical abstract

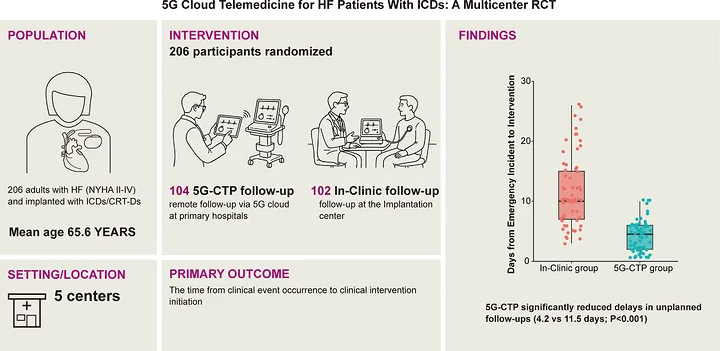

## 1 Introduction

Heart failure (HF) imposes a substantial clinical and economic burden [1], affecting an estimated 64 million people worldwide, with prevalence increasing because of population aging. Implantable cardioverter‐defibrillators (ICDs) and cardiac resynchronization therapy defibrillators (CRT‐Ds) are cornerstone therapies for patients with chronic HF who meet guideline‐directed indications [2, 3, 4], significantly reducing mortality and sudden cardiac death. However, the long‐term benefits of these cardiac implantable electronic devices (CIEDs) depend on rigorous postoperative follow‐up to monitor device function, detect arrhythmic events, and optimize therapeutic parameters [5]. Current guidelines recommend outpatient visits every 3–6 months [6, 7], but real‐world compliance remains suboptimal, particularly in geographically dispersed populations.

Postoperative follow‐up for patients with ICDs/CRT‐Ds faces three interrelated challenges. First, ICD/CRT‐D programming is highly complex, requiring electrophysiology‐trained physicians who can interpret intracardiac electrograms, optimize pacing and defibrillation parameters, and troubleshoot device algorithms. Such expertise is concentrated in urban tertiary centers and remains largely unavailable at community hospitals [8, 9, 10]. Second, rising ICD/CRT‐D implantation volumes are straining specialized follow‐up clinics, with prolonged appointment wait times and limited time per patient for comprehensive device assessment. Third, delayed detection of critical events remains a major concern: conventional follow‐up relies on scheduled every 3–6 months or symptom‐driven visits, risking substantial delays between silent but clinically significant events (e.g., lead dysfunction, arrhythmic episodes) and clinical recognition. The TRUST study underscored this inefficiency, revealing that only 6% of scheduled In‐clinic visits identified actionable CIED events [11]. Patients in remote or underserved regions also face geographical and economic barriers, that often reduce follow‐up adherence and compromise outcomes [12, 13, 14].

Remote monitoring (RM) has advanced substantially since the 1970s [15], from early transtelephonic systems that enabled basic parameter transmission [16, 17] to modern platforms providing automatic data collection and real‐time alerts for device integrity issues and arrhythmic events [18, 19, 20]. Randomized trials such as CONNECT and EVOLVO showed that RM reduced time to clinical decision‐making and healthcare utilization [21, 22, 23]. However, conventional RM cannot perform remote device reprogramming. When parameter adjustments are required, patients must still undertake in‐person clinic visits, a major bottleneck for those residing far from specialized centers. This creates a gap between monitoring and intervention where timely detection is not matched by timely action.

Recent advances in 5G telecommunications and cloud computing offer a practical solution to this monitoring‐intervention gap. The 5G‐cloud technology platform (5G‐CTP) leverages ultra‐low latency and high‐reliability networks to allow physicians to perform remote device interrogation, testing, and reprogramming in real time. Under this model, patients visit nearby community hospitals where local physicians connect the device to a 5G terminal; remote specialists then access the cloud‐based system to perform real‐time interrogation, reprogramming, and clinical decision‐making. This model eliminates the need for long‐distance travel to implantation centers, enables timely intervention for urgent events, and leverages existing community healthcare infrastructure while maintaining specialist‐level care.

Despite the promising potential of 5G‐CTP, existing evidence is limited to small‐scale studies confirming its technical feasibility and safety [24, 25, 26]. Robust randomized controlled trials evaluating whether this platform shortens the time from clinical event detection to intervention remain lacking. Such timeliness represents its core advantage over conventional remote monitoring. This evidence gap is clinically consequential, as prolonged delays in device reprogramming and other therapeutic adjustments for HF patients in remote areas result in avoidable hospitalizations and worsening outcomes. The “Make Changes” study was a prospective, multicenter, randomized controlled trial to evaluate whether 5G‐CTP follow‐up reduced the time from clinical event occurrence to intervention compared with standard In‐clinic care in HF patients with ICDs/CRT‐Ds.

## 2 Results

### 2.1 Baseline Characteristics

Between October 2022 and February 2024, 230 patients were enrolled from five participating centers (as detailed in the Methods section). Of these, 206 met the inclusion criteria, and were randomly assigned to the 5G‐CTP group (*n* = 104) or the In‐Clinic group (*n* = 102). The trial flowchart is shown in Figure S1. Baseline demographic characteristics, clinical parameters, and medication use were comparable between groups (Table 1). Implanted devices included single‐chamber ICDs in 90 patients (44%), dual‐chamber ICDs in 22 (11%), and CRT‐D devices in 94 (45%). The primary implantation indication was primary prevention in 46% of patients with ICDs.

**TABLE 1:** Demographics and baseline clinical parameters.

	All (*n* = 206)	In‐Clinic group (*n* = 102)	5G‐CTP group (*n* = 104)	*p* value
Male gender, *n* (%)	153 (74.27)	73 (71.57)	80 (76.92)	0.3794
Ages, years	65.57 (11.19)	65.13 (10.69)	66.01 (11.70)	0.5729
Follow up distance (One‐Way, km)	186.5 (41.0, 372.0)	184.5 (45.0, 364.0)	187.0 (40.0, 382.5)	0.9748
MLHFQ Score	46.59 (15.80)	47.01 (15.62)	46.18 (16.03)	0.7081
**Underlying heart disease, *n* (%)**				
Ischemic	58 (28.16)	23 (22.55)	35 (33.65)	0.0764
Dilated CMP	89 (43.20)	48 (47.06)	41 (39.42)	0.2687
Hypertrophic CMP	10 (4.85)	5 (4.90)	5 (4.81)	1.0000
Valvular	17 (8.25)	11 (10.78)	6 (5.77)	0.1909
Other	33 (16.02)	15 (14.71)	18 (17.31)	0.6107
**NYHA class, *n* (%)**				0.8960
II	48 (23.30)	23 (22.55)	25 (24.04)	
III	126 (61.17)	62 (60.78)	64 (61.54)	
IV	32 (15.53)	17 (16.67)	15 (14.42)	
Primary prevention, *n* (%)	95 (46.12)	50 (49.02)	45 (43.27)	0.4078
Single chamber ICD, *n* (%)	90 (43.69)	40 (39.22)	50 (48.08)	0.1998
Dual chamber ICD, *n* (%)	22 (10.68)	11 (10.78)	11 (10.58)	0.9616
CRT defibrillator, *n* (%)	94 (45.63)	51 (50.00)	43 (41.35)	0.2125
Myocardial infarction, *n* (%)	24 (11.65)	10 (9.80)	14 (13.46)	0.4133
Previous CABG or angioplasty, *n* (%)	51 (24.76)	22 (21.57)	29 (27.88)	0.2937
Previous valve surgery, *n* (%)	12 (5.83)	7 (6.86)	5 (4.81)	0.5289
AF persistent, *n* (%)	27 (13.11)	15 (14.71)	12 (11.54)	0.5006
Hypertension, n (%)	91 (44.17)	41 (40.20)	50 (48.08)	0.2548
Diabetes mellitus, *n* (%)	48 (23.30)	21 (20.59)	27 (25.96)	0.3617
Chronic kidney disease, *n* (%)	17 (8.25)	10 (9.80)	7 (6.73)	0.4229
COPD, *n* (%)	1 (0.49)	0	1 (0.96)	1.0000
LV ejection fraction, *n* (%)	40.78 (13.67)	40.17 (14.11)	41.39 (13.27)	0.5250
**Medications, *n* (%)**				
β‐blocker	139 (67.48)	71 (69.61)	68 (65.38)	0.5177
ACE inhibitor/ARB	17 (8.25)	7 (6.86)	10 (9.62)	0.4728
ARNI	137 (66.50)	66 (64.71)	71 (68.27)	0.5880
Diuretic	120 (58.25)	63 (61.76)	57 (54.81)	0.3114
Antisterone	123 (59.71)	66 (64.71)	57 (54.81)	0.1476
Dapagliflozin/Empagliflozin	75 (36.41)	36 (35.29)	39 (37.50)	0.7422
Class III antiarrhythmic	45 (21.84)	22 (21.57)	23 (22.12)	0.9243
Anticoagulant, *n* (%)	50 (24.27)	25 (24.51)	25 (24.04)	0.9371
Antiplatelet, *n* (%)	53 (25.73)	25 (24.51)	28 (26.92)	0.6920

*Note*: Age and MLHFQ Score reported as Mean (SD). Distance reported as Median (Interquartile Range).

Abbreviations: AF, Atrial Fibrillation; ACE, angiotensin‐converting enzyme; CABG, coronary artery bypass grafting; COPD, chronic obstructive pulmonary disease; CMP, cardiomyopathy; CRT, cardiac resynchronization therapy; LV, left ventricular; MLHFQ, Minnesota Living with Heart Failure Questionnaire; NYHA, New York Heart Association.

The median follow‐up was 12.0 months. Five patients in the 5G‐CTP group and seven in the In‐Clinic group were lost to follow‐up. In the 5G‐CTP group, two patients required electrode repositioning because of dislocation or elevated thresholds. In the In‐Clinic group, one patient underwent ICD electrode removal and reimplantation because of lead wear, and one required electrode repositioning for an elevated threshold. Two deaths occurred during the study: one sudden cardiac death in the 5G‐CTP group and one HF‐related death in the In‐Clinic group.

During the study, 427 remote connections were completed. Six (1.4%) were interrupted after successful establishment, with all devices automatically reverting to baseline settings within 580–1080 ms. The overall success rate was 98.6%, and no procedure‐related complications were observed.

### 2.2 Response Time for Adverse Events

By March 2025, when the study ended, 142 unplanned follow‐up events were recorded. 72 events in 56 patients in the 5G‐CTP group, and 70 events in 57 patients in the In‐Clinic group. Baseline characteristics of patients with events were comparable (Table S1). The most common events were acute HF exacerbations (*n* = 72, 50.7%), rapid ventricular rates (mean ≥100 bpm) during atrial tachycardia/atrial fibrillation (AT/AF) (*n* = 35, 24.6%), and shock delivery in ≥1 episode (*n* = 29, 20.4%) (Table 2).

**TABLE 2:** Days from a clinical/alert event to a clinical intervention.

	Events No. (Patients No.)		Least Squares mean ± SD			
Clinical/Alert Event	In‐Clinic group	5G‐CTP group	In‐Clinic group	5G‐CTP group	Least Squares mean difference (95% CI)	*p* (Mixed Model)
Acute heart failure	37 (35)	35 (35)	12.7 ± 0.59	5.5 ± 0.59	−7.2 (‐8.8, ‐5.5)	<0.001
Fast V rate during AT/AF: Mean V rate of at least 100 bpm	11 (10)	11 (10)	13.1 ± 1.54	4.7 ± 1.54	−8.4 (‐12.9, ‐3.8)	0.001
Shock delivered in an episode (at least one)	14 (13)	15 (15)	5.6±0.34	1.4±0.33	−4.2 (‐5.2, ‐3.3)	<0.001
Total	65 (56)	64 (54)	11.5±0.58	4.2±0.59	−7.3 (‐8.9, ‐5.7)	<0.001

Abbreviations: AT/AF, atrial tachycardia/atrial fibrillation; VT/VF, ventricular fibrillation.

Among unplanned follow‐ups, 129 events (90.8%) required clinical intervention, including 64 (49.6%) in the 5G‐CTP group and 65 (50.4%) in the In‐Clinic group. Acute HF exacerbation was the leading cause of intervention (35 [48.6%] vs. 37 [52.8%]). Most interventions involved device reprogramming (35 [44.8%] vs. 37 [43.5%]) or medication adjustment (29 cases [37.1%] vs. 30 [35.2%]) (Table S2). The mean time from event to intervention was significantly shorter in the 5G‐CTP group than in the In‐Clinic group (4.2±0.59 vs. 11.5 ± 0.58 days, p<0.001) (Figure 1A). The device programming time per patient for unscheduled visits is shown in Figure 1B, with no significant difference between the 5G‐CTP and In‐Clinic groups. Subgroup analysis showed superior performance of 5G‐CTP in all subgroups except “persistent atrial fibrillation” (*p* = 0.0667) (Figure S2).

**FIGURE 1:**
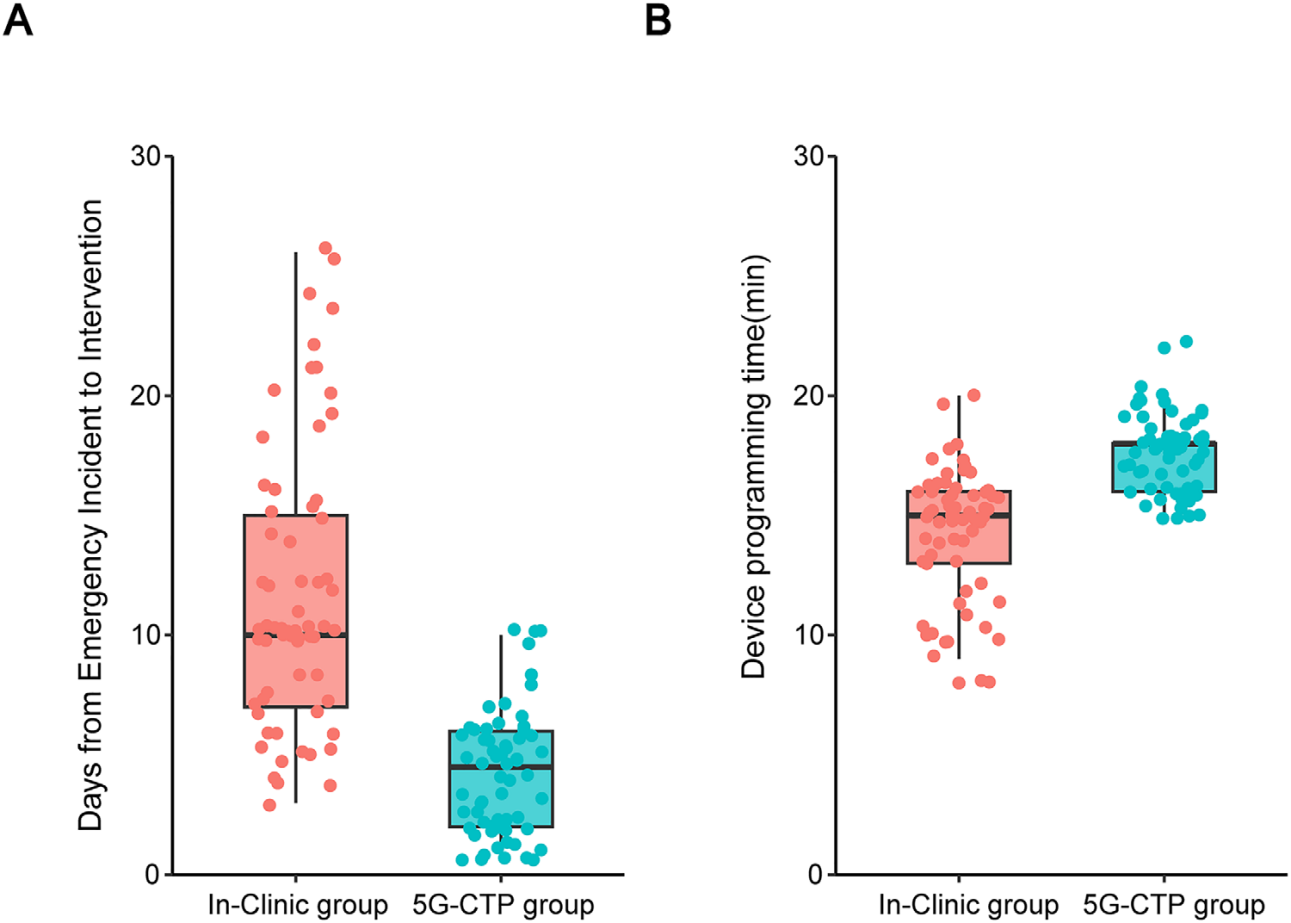
Comparative analysis of time distribution patterns for unscheduled patient visits in the In‐Clinic and 5G‐CTP groups. (A) Time from clinical event to intervention per patient in two arms (57 patients in In‐Clinic and 56 in 5G‐CTP) for unscheduled visits. (B) Programmed time per patient in the two groups for unscheduled visits. Red bars indicate the In‐Clinic group; green bars indicate the 5G‐CTP group.

During scheduled follow‐up, 413 events were identified: 216 (52.3%) in the 5G‐CTP group and 197 (47.7%) in the In‐Clinic group. Baseline characteristics were comparable. The most common events were reduced device‐detected thoracic impedance (CorVue) (*n* = 151, 36.6%) and increased AT/AF burden (*n* = 146, 35.4%). Physicians intervened in 160 events (38.7%), including 83 (51.9%) in the 5G‐CTP group and 77 (48.1%) in the In‐Clinic group. Interventions were primarily triggered by reduced CorVue thresholds (74 [34.2%] vs. 77 [39.0%]) and AT/AF burden lasting ≥6 h (83 [38.4%] vs. 63 [31.9%]). Medication adjustment was the most common intervention (Table S3). Response times did not differ significantly between groups during scheduled follow‐up.

### 2.3 Healthcare Resource Utilization

A total of 115 patients required emergency department visits (8 [7.6%] in the 5G‐CTP group vs. 7 [6.8%] in the In‐Clinic group) or unplanned outpatient visits (53 [50.9%] vs. 47 [46.0%]) because of HF, arrhythmias, or ICD‐related events. In addition, 21 patients were hospitalized overnight for worsening HF (9 [8.6%] vs. 12 [11.7%]), and three were hospitalized for arrhythmias (1 [0.9%] vs. 2 patients [1.9%]). Rates of emergency department visits, unplanned outpatient visits, and hospitalizations for HF, arrhythmias, or ICD‐events (Table S4). However, the 5G‐CTP group had shorter hospital stay than the In‐Clinic group (4.18 ± 0.98 vs. 5.19 ± 1.38 days, *p* = 0.047) (Figure 2).

**FIGURE 2:**
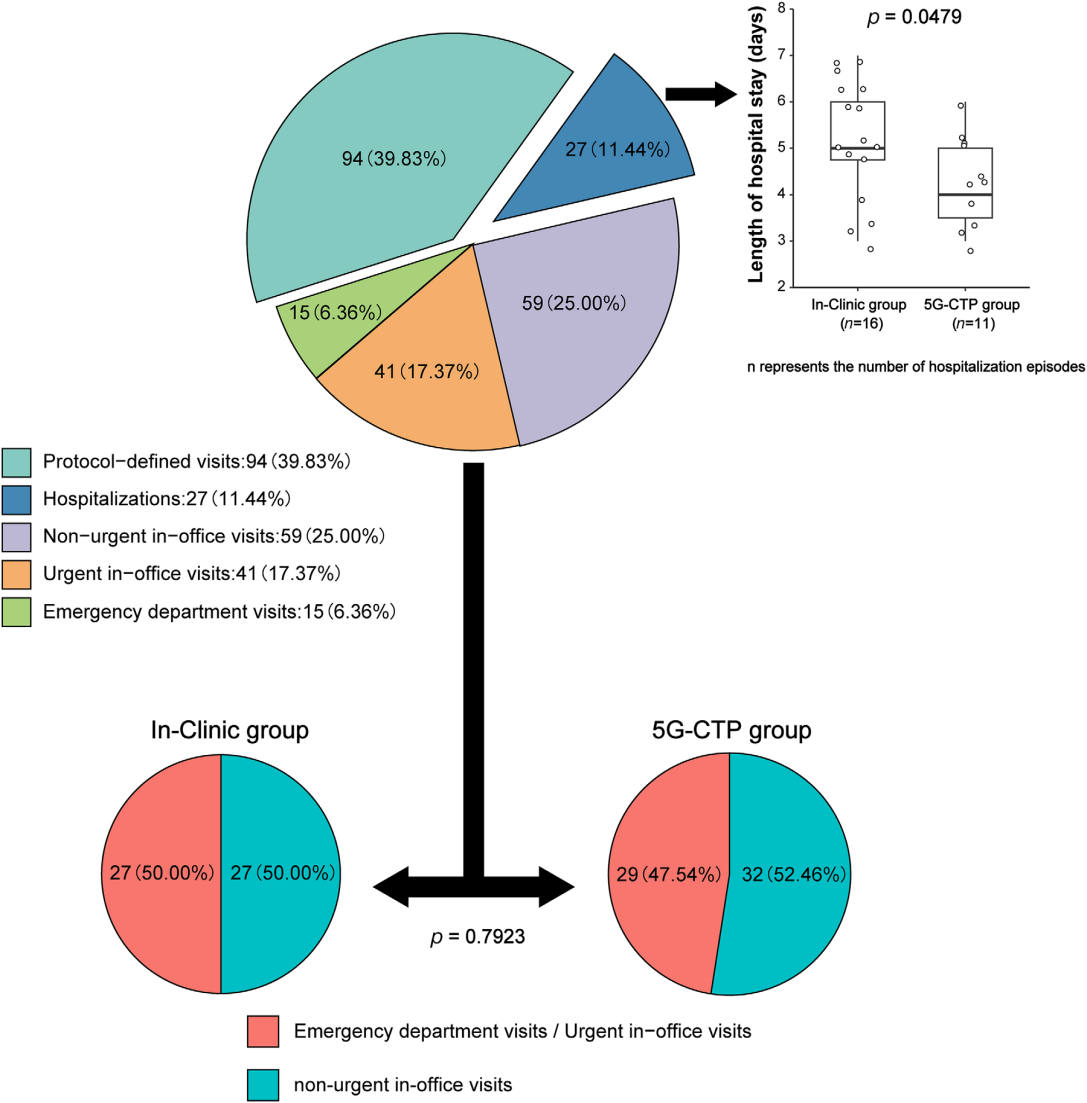
Proportion of medical resource utilization. Annual rates by randomization group of scheduled visits (protocol‐required in‐office visits), Urgent in‐office visits (patient‐initiated, non‐protocol visits), non‐urgent in‐office visits (non‐protocol, patient‐initiated visits), emergency room admissions, and hospitalizations (bar graph showing length of hospital stay for each randomization group).

### 2.4 Quality of Life

Quality of life was assessed using the Minnesota Living with Heart Failure Questionnaire (MLHFQ). Baseline scores were similar between the 5G‐CTP and In‐Clinic groups (46.18 ± 1.55 vs. 47.01 ± 1.57). After 6 months, changes in MLHFQ scores remained comparable (44.36 ± 1.17 vs. 45.88 ± 1.18). At 12 months, however, the 5G‐CTP group showed greater improvement than the In‐Clinic group (37.99 ± 1.29 vs. 42.64 ± 1.31; *p* = 0.012) (Figure S3).

## 3 Discussion

This study introduces 5G‐CTP as a novel postoperative management model for patients with ICD/CRT‐D. The “Make Changes” study, the first RCT, compared the effectiveness and timeliness of 5G‐CTP follow‐up with traditional outpatient follow‐up. The study findings show that 5G‐CTP significantly reduced the mean time from event detection to clinical intervention and shortened hospital stays compared with conventional follow‐up, while improving postoperative quality of life as reflected by lower MLHFQ scores.

According to the national Direct Reporting System for Interventional Treatment of Arrhythmia in China, 6547 ICDs and 3508 CRT‐Ds were implanted in 2021, representing year‐on‐year increases of 36.0% and 36.9%, respectively [27]. Most implantations occur in urban tertiary hospitals, whereas rural areas account for only a small fraction of the national total. As implantation rates rise, demand for postoperative follow‐up also increases, with traditional In‐clinic visits remaining the primary approach in China. However, patient adherence is suboptimal, with many patients failing to complete formal follow‐up within the recommended 2–12 weeks after CIED implantation. Patients in remote areas face challenges related to uneven resource distribution, financial costs, and geographic barriers. Patients with newly implanted ICDs/CRT‐Ds frequently experience surgery or management‐related adverse events [28]. Studies such as EVOLVO and MORE‐CARE phase 1 reported high rate of emergency follow‐up, primarily HF and AF [21, 29]. Similarly, most emergency events in this study, involved HF, ventricular tachycardia (VT), and AF, and were managed through device reprogramming and treatment adjustments.

In recent years, guideline‐recommended RM has enabled real‐time monitoring of ICD/CRT‐D devices. The Connect trial showed that RM accelerated clinical decision‐making by 17.4 days compared with in‐office follow‐up, although response times varied (0‐13 days) because of patient‐related issues (e.g., device failure) and clinician response variability [30]. However, device reprogramming still requires in‐clinic visits, potentially delaying treatment for patients in remote areas. The 5G‐CTP follow‐up model optimizes resource use by enabling remote consultation and programming through a 5G cloud platform at nearby hospitals. This approach supports urgent local intervention while substantially reducing travel time. In this study, a patient with an ICD located 2000 km away received successful remote intervention for a VT storm through the 5G platform (see Supporting Information Video). Among unplanned follow‐ups for urgent events, the 5G‐CTP group achieved a 36% shorter mean time from event detection to clinical decision than the conventional follow‐up group (4.2 vs. 11.5 days, *p*<0.001).

Patient safety is essential in both hospital‐based and 5G‐CTP follow‐up for patients with ICD/CRT‐D and depends on device performance (e.g., battery status and lead integrity) and patient condition. In this study, one patient in the 5G‐CTP group experienced early lead displacement, whereas one outpatient developed late‐stage lead wear. Both sought medical attention after ICD impedance alarms, but lead revision occurred sooner in the 5G‐CTP group (3 vs. 10 days). The 5G‐CTP group also showed faster intervention for HF and major arrhythmias, including HF (5.5 vs. 12.7 days), AF with abnormal ventricular rates (4.7 vs. 13.1 days), and VT (1.4 vs. 5.6 days). Although RM promptly alerts physicians, it cannot prevent all adverse events, because some conditions are unpredictable. Thus, RM cannot fully replace in‐person follow‐up, whereas 5G‐CTP combines remote convenience with clinical safety.

This study also compared healthcare utilization between patients receiving 5G‐CTP and standard care. No significant differences were observed in healthcare utilization, including scheduled or unscheduled outpatient visits, and emergency department visits. However, the 5G‐CTP group had shorter hospital stays, suggesting that earlier intervention reduced hospitalization. Overall, 5G‐CTP was as safe as traditional follow‐up while improving intervention timeliness and patient outcomes. The American Heart Association emphasizes that patient‐reported outcomes are essential for improving care quality and evaluating treatment effects [31, 32]. Consistent with this recommendation, patients using 5G‐CTP reported better quality of life after one year, likely because remote follow‐up enabled more timely clinical decision‐making.

## 4 Limitation

Currently, 5G terminals are compatible only with Abbott programmers, limiting this study to Abbott ICD/CRT‐D devices. Additional randomized controlled trials are needed to evaluate the effectiveness of 5G‐CTP with other device manufacturers. As an open‐label trial, this study could not blind participants or investigators to group assignment. We acknowledge that this may have introduced performance and reporting bias. In particular, patients in the 5G‐CTP group, aware of being allocated to a novel remote care model, may have reported more favorable MLHFQ scores at 12 months due to heightened engagement, differential expectations, or a placebo‐like effect, rather than solely due to the intervention itself. This limitation should be considered when interpreting the quality‐of‐life findings. In addition, although 5G‐CTP follow‐up improves convenience, it does not reduce outpatient visits because patients still require hospital evaluation for symptoms or device alerts. However, it reduces travel time and costs, which may offset outpatient expenses. Larger studies are needed to determine its cost‐effectiveness for postoperative ICD/CRT‐D follow‐up. Finally, unlike traditional RM, 5G‐CTP does not provide real‐time monitoring, which may delay detection of asymptomatic events (e.g., arrhythmias). Randomized RM studies have shown that detecting these events increases unscheduled visits and HF hospitalizations [11, 29, 33], although adverse event rates remain comparable with traditional follow‐up [34] This proof‐of‐concept trial demonstrates that 5G‐CTP is feasible in significantly reducing the time interval from clinical event onset to intervention. These urgent follow‐ups are clinically meaningful, as earlier intervention is hypothesized to translate into improved clinical outcomes. Indeed, the 5G‐CTP group showed a shorter mean hospitalization duration than the In‐Clinic group (4.2 vs.5.2 days; *p* = 0.047); however, the absolute difference of approximately 1 day is modest, warranting cautious interpretation. Whether the timeliness advantage of 5G‐CTP intervention translates into improvements in hard clinical endpoints, including cardiovascular mortality, remains to be established in larger, randomized controlled trials.

## 5 Conclusions

This study demonstrates that 5G‐CTP is feasible postoperative follow‐up strategy for HF patients with ICDs/CRT‐Ds. It is particularly beneficial in resource‐limited regions by reducing time and financial burdens while improving access to specialist care and patient adherence. The “Make Changes” study further supports that 5G‐CTP is a promising approach for postoperative ICD/CRT‐D management in patients with HF.

## 6 Methods

### 6.1 Study Design and Participants

The “Make Changes” study was a prospective, multicenter, randomized controlled trial (RCT) of patients with HF who underwent ICD or CRT‐D implantation. Because the 5G follow‐up terminal is compatible only with Abbott programmers, all enrolled patients received Abbott devices (ICD models: CD1377‐36C, CD1377‐36QC, CD2377‐36C, CD2377‐36QC; CRT‐D models: CD3371‐40, CD3371‐40Q). Eligible patients had New York Heart Association (NYHA) functional class II–IV and were consecutively recruited from Changhai Hospital, Shanghai Chest Hospital, Fujian Medical University Union Hospital, Qingdao 971 Hospital, and Xinjiang Military Region General Hospital (Figure 3). Patients were randomly assigned in a 1:1 ratio to either the 5G‐CTP group or the In‐Clinic group using permuted block randomization with a fixed block size of 4, stratified by enrollment center. The randomization sequence was generated by an independent statistician prior to study initiation. Allocation concealment was ensured using sequentially numbered, opaque, sealed envelopes, which were opened by the investigators responsible for enrolling participants only after confirming the participant's eligibility for enrollment.

**FIGURE 3:**
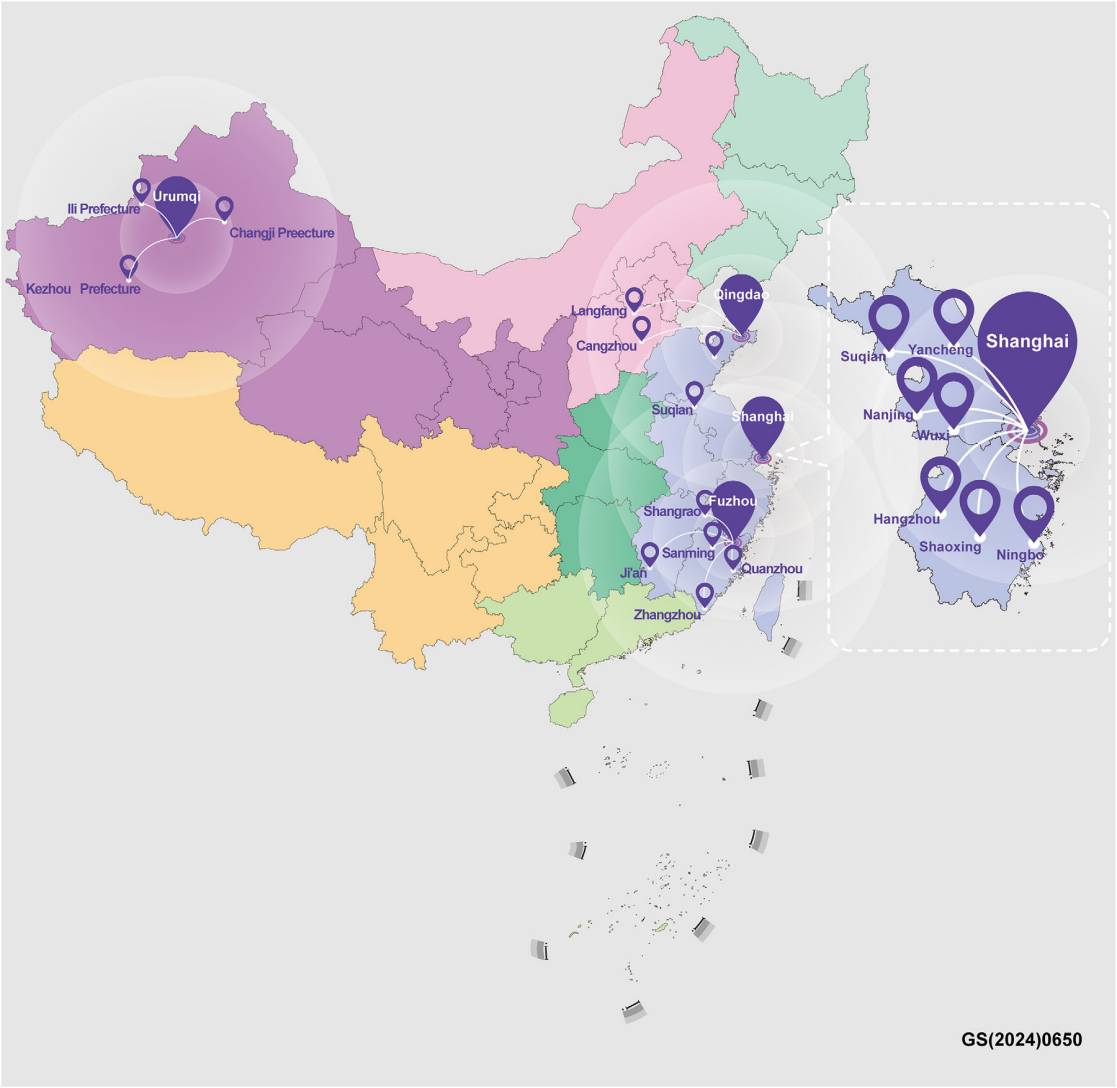
Heart failure patients newly implanted with ICD/CRTD from five implantation centers across four districts (Shanghai, Fuzhou, Qingdao, and Urumqi) were enrolled in this study. These centers used a 5G cloud platform to conduct follow‐up care for patients from surrounding cities.

Inclusion criteria were: (1) successful ICD/CRT‐D implantation; (2) residence ≥50 km from the implantation center or follow‐up travel time ≥1 h; (3) access to a 5G‐enabled hospital within 50 km or ≤1 h for follow‐up; and (4) ability to independently complete the MLHFQ. Exclusion criteria were: (1) age <18 years; (2) acute myocardial infarction within the previous 90 days; (3) current or planned enrollment in another trial affecting study endpoints; (4) physician‐estimated life expectancy <1 year; (5) pregnancy or lactation; (6) cognitive impairment preventing independent questionnaire completion; or (7) inability or unwillingness to provide written informed consent. This study was conducted at five centers with their affiliated network hospitals, with institutional ethics committee approval. Written informed consent was obtained from all patients before enrollment.

### 6.2 Follow‐up

Patients in the 5G‐CTP group attended outpatient visits at the implantation center, at baseline and 12 months and underwent additional 5G‐CTP follow‐up at primary hospitals (without local ICD/CRTD follow‐up capability) at 3, 6, and 9 months. Through 5G‐CTP, the five centers established a regional cloud‐based follow‐up network linking primary hospitals, enabling one‐to‐one and one‐to‐many telemonitoring. Primary hospitals were equipped with a CIED programmer and a 5G remote support terminal (Figure 4). In contrast, patients in the In‐Clinic group attended in‐person follow‐up at the implantation center at baseline and 3, 6, and 9 months. Previous studies have shown that ICD/CRTD audio alerts improved clinical event detection, although many detected events were asymptomatic or nonurgent [35]. To reduce anxiety and unnecessary visits, noncritical alerts, excluding lead and device‐related warnings, were disabled.

**FIGURE 4:**
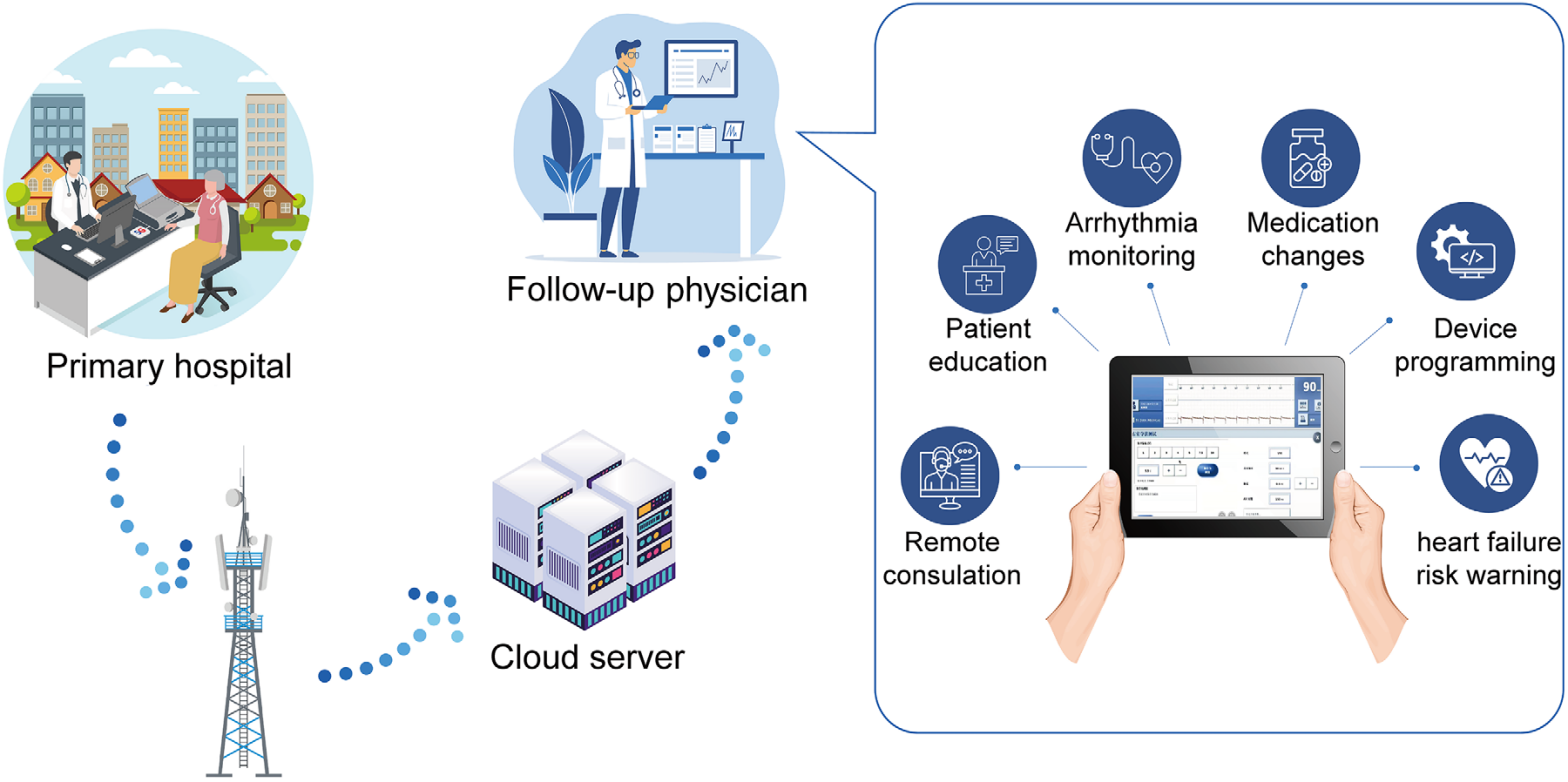
Depiction of follow‐up model for patients in the 5G‐CTP group. Doctors at primary healthcare institutions connect the 5G‐supported terminal to the programmer and place the programming probe on the patient's ICD/CRTD device. Subsequently, they notify remote follow‐up experts via phone calls or text messages. Upon receiving the notification, remote follow‐up experts log in to the Cloud Follow‐up APP using tablets or mobile phones to establish a remote connection with the 5G‐supported terminal on the patient's end. Follow‐up experts can conduct real‐time inquiries, tests, and reprogramming operations on the ICD/CRTD device according to actual needs. Meanwhile, based on the assessment results of the patient's condition and the device, they implement corresponding intervention measures (such as health education, medication adjustments, etc.). The entire remote business system is deployed on cloud servers and employs multi‐layer protection mechanisms including firewalls, customized antivirus software, vulnerability scanning, and intrusion detection.

During 5G‐CTP follow‐up, patients visited a primary hospital where a physician placed a programmer probe connected to a 5G terminal on the CIED and notified the remote specialist by phone or SMS. The specialist accessed the 5G Cloud Follow‐Up APP on a mobile device using two‐factor authentication (username/password plus verification code) and remotely interrogated and reprogrammed the CIED through the 5G platform. If communication failed, the CIED automatically reverted to its baseline settings. The cloud‐based system recorded session times and programming parameters for audit purposes. Hosted on secure cloud servers with multilayer security, including firewalls, antivirus, software, and routine security scans, the system ensured data privacy. Trained personnel were available for emergency support, to ensure network reliability and procedural safety.

### 6.3 Observation Endpoints

The primary objective was to compare the time from clinical event occurrence to initiation of clinical intervention between the 5G‐CTP and In‐Clinic groups. Clinical events included device‐related events (e.g., abnormal lead impedance, CRT% <90%, defibrillator lead impedance abnormalities, prolonged charging time, backup VVI mode activation, and pocket or device infection) and disease‐related events (e.g., increased AF burden, ventricular rate >100 bpm during AF, worsening HF, VT/VF events, shock/ATP therapy, and CorVue alerts). Only events requiring clinical intervention were included in the primary endpoint analysis.

Clinical intervention was defined as pharmacological treatment modification (e.g., initiation or adjustment of HF or antiarrhythmic therapy), device reprogramming (e.g., increasing pacing output >1 V or modifying algorithms/settings to improve VT/supraventricular tachycardia detection), or planned hospitalization (e.g., cardioversion or ablation). Detailed flowcharts guided interventions for AT/AF burden (Figure S4) and CorVue alerts (Figure S5) to ensure consistent clinical decision‐making. Secondary objectives were to compare the proportions of patients requiring emergency or unplanned outpatient visits for HF, arrhythmia, or ICD‐related events, and to evaluate quality of life using the MLHFQ [36].

### 6.4 Data Collection

Clinical and device data were collected during both scheduled and unscheduled in‐clinic or remote visits. Event detection time was defined as the time when alert criteria were met. Event resolution was defined as the date of physician intervention based on stored device data and was used to calculate the interval from event occurrence to clinical intervention. Events confirmed without subsequent clinical intervention were excluded from the primary endpoint analysis. An independent Clinical Event Committee comprising three physicians not involved in the trial adjudicated all disputed events.

### 6.5 Sample Size Calculation

Referencing data from authoritative remote monitoring trials [29, 30], we set the 5G‐CTP group mean response time at 6 days (SD 4.9) and the conventional follow‐up group at 22 days (SD 29.9). With a two‐sided α of 0.05 and 90% power, the initial calculation required 40 subjects per group. Considering that approximately 50% of patients experienced at least one event, as reported in Lazarus et al.’s global CIED database of 11,624 patients [18]. We calculated 80 subjects per group to cover the target events, with an additional 20% reserve for anticipated dropout, resulting in a final target of 100 subjects per group.

### 6.6 Statistical Analysis

All analyses were performed in the intention‐to‐treat population. Continuous variables are presented as mean ± SD for normally distributed data, or median (interquartile ranges) for nonnormally distributed data. Categorical variables were presented as frequencies and percentages. Between‐group comparisons were performed using the chi‐squared test, *t*‐test, or Wilcoxon rank‐sum test, as appropriate.

For the primary outcome (time from clinical event to intervention), mixed‐effects models estimated the least squares mean difference and 95% confidence interval (CI) between groups, with patients and centers treated as random effects. The same approach was used to compare programming times. Because MLHFQ scores were measured repeatedly, mixed‐effects models with a group‐by‐time interaction were used to assess longitudinal changes and estimate the least squares mean difference and 95% CI at 12 months. Missing data resulting from loss to follow‐up were not imputed.

Subgroup analyses of the primary outcome were performed according to center, sex, age (<60 vs. ≥60 years), NYHA class, ischemic cardiomyopathy, baseline left ventricular ejection fraction (≤35% vs. >35%), persistent AF, device type, and primary ICD prevention. Statistical analyses were performed using R version 4.0.3 and SAS version 9.4 (SAS Institute Inc., Cary, NC). A two‐tailed *p* < 0.05 was considered statistically significant.

## Author Contributions

Zhongkai Wang had full access to all data in the study and takes responsibility for the integrity of the data and the accuracy of the data analysis. *Concept and design*: Pan Li and Fayuan Fu. *Acquisition, analysis, or interpretation of data*: Yiding Qi and Bo Liu. *Drafting of the manuscript*: Pan Li and Zhongkai Wang. *Critical review of the manuscript for important intellectual content*: Zhifu Guo and Jingjuan Huang. *Statistical analysis*: Jing Jiang and Guowei Wang. *Obtained funding*: Fayuan Fu, Zhongkai Wang, and Pan Li. *Administrative, technical, or material support*: Xianxian Zhao. *Supervision*: Xinghua Shan and Jingjuan Huang. All authors have read and approved the final manuscript.

## Conflicts of Interest

The authors declare no conflicts of interest.

## Funding

This work was supported by the Fujian Provincial Science and Technology Department's guiding projects (grant no.: 2020Y0033), the Shanghai Science and Technology Innovation Action Plan (grant no.: 22Y11909400), and the Shanghai Oriental Talents Program Youth Project (grant no.: QNJY2024060).

## Ethics Statement

This study was conducted in accordance with the principles of the Declaration of Helsinki and was approved by the Research Ethics Committee of Changhai Hospital, Naval Medical University (ethics approval number: CHEC2022‐201). The trial was registered with the Chinese Clinical Trial Registry (ChiCTR2200059172). Written informed consent was obtained from all participants.

## Supporting information




**Supporting File 1**: mco271037‐sup‐0001‐SuppMat.docx

**Supporting File 2**: mco271037‐sup‐0002‐VideoS1.mp4

## Data Availability

One year following the publication of the study, de‐identified trial data will be made available upon request to the corresponding author (wangzksmmu@163.com). Requesters are required to clearly outline the specific purpose for which the data will be used in their request. In addition, a signed data access agreement will be mandatory for data access.
