# Supplementary material for: 5G Cloud‐Enabled Telemedicine for Remote Management of Heart Failure Patients With Implantable Cardioverter‐Defibrillators: A Multicenter Open‐Label Randomized Controlled Trial
Source: MedComm (2020). 2026 Sep 29;7(10):e71037. doi: 10.1002/mco2.71037 (PMC13620867; doi:10.1002/mco2.71037)
Supplement: Supplementary file 1 [file MCO2-7-e71037-s002.docx]

5G Cloud-Enabled Telemedicine for Remote Management of Heart Failure Patients With Implantable Cardioverter-Defibrillators: A Multicenter Open-Label Randomized Controlled Trial

Pan Li,^1#^ Fayuan Fu,^2^^#^ Yiding Qi,^3#^ Xianxian Zhao,^1^ Yingyi Qin,^4^ Xinghua Shan,^1^ Jing Jiang,^5^ Guowei Wang,^6^ Jingjuan Huang,^3*^ Bo Liu,^1,7*^ Zhifu Guo,^1*^ Zhongkai Wang^1*^

^1^Department of Cardiology, Changhai Hospital, Naval Medical University, 168 Changhai Rd, Shanghai, 200433, China.

^2^Department of Cardiology, Union Hospital, Fujian Medical University, 29 Xinquan Rd, Fuzhou, 350001, China.

^3^Department of Cardiology, Shanghai Chest Hospital, Shanghai Jiao Tong University School of Medicine, 241 Huaihai Rd, Shanghai, 200030, China.

^4^Department of Health Statistics, Naval Medical University, 800 Xiangyin Rd, Shanghai, 200433, China.

^5^Department of Cardiology, PLA Navy No. 971 Hospital, 22 Minjiang Rd, Qingdao, 266071, China.

^6^Department of Cardiology, Xinjiang Military Region General Hospital, 359 Youhao South Rd, Urumqi, 830000, China.

^7^Department of Cardiology, Xinhua Hospital, Shanghai Jiao Tong University School of Medicine, 1665 Kongjiang Rd, Shanghai, 200092, China.

^#^These authors contributed equally to this work.

^*^Corresponding author. Email: wangzksmmu@163.com (ZK. W); [guozhifu@126.com](mailto:guozhifu@126.com) (ZF. G); [liubo@xinhuamed.com.cn](mailto:liubo@xinhuamed.com.cn) (B.L). [hjjxinhua@163.com](mailto:hjjxinhua@163.com) (JJ. H)

**Supplementary Figure legend.**

Figure S1. Flowchart depicting study follow-up experiences.

Figure S2. Forest plot subgroup analysis assessing the temporal impact of different clinical characteristics on 5G-CTP or In-Clinic follow-up approaches.

Figure S3. Temporal trends in Mean MLHFQ Scores over time in the In-Clinic and 5G-CTP groups. Red bars indicate the In-Clinic group; Blue bars indicate the 5G-CTP group. MLHFQ, Minnesota Living with Heart Failure Questionnaire.

Figure S4. Flowchart illustrating AT/AF burden episodes.

Figure S5. Flowchart for the management of fluid accumulation alerts (CorVue).

**Supplementary video.** A patient with an ICD located 2000 km away received successful remote intervention for a VT storm through the 5G platform.

**Figure S1.**

**Figure S2.**

**Figure S3.**

**Figure S4.**

**Figure S5.**

## **Table S1.** Comparison of baseline characteristics between two groups of patients with unplanned visits.

| Item | All (n = 113) | In-Clinic group  (n = 57) | 5G-CTP group  (n = 56) | *P* |
| --- | --- | --- | --- | --- |
| Male gender, n (%) | 88(77.88) | 44(77.19) | 44(78.57) | 0.8599 |
| Ages, years | 64.71(10.59) | 64.88(10.07) | 64.54(11.17) | 0.8648 |
| Underlying heart disease, n (%) |  |  |  |  |
| Ischemic | 35(30.97) | 13(22.81) | 22(39.29) | 0.0582 |
| Dilated CMP | 51(45.13) | 31(54.39) | 20(35.71) | 0.0461 |
| Hypertrophic CMP | 2(1.77) | 1(1.75) | 1(1.79) | 1.0000 |
| Valvular | 10(8.85) | 4(7.02) | 6(10.71) | 0.7184 |
| Other | 16(14.16) | 8(14.04) | 8(14.29) | 0.9695 |
| NYHA class, n (%) |  |  |  | 0.8604 |
| II | 22(19.47) | 10(17.54) | 12(21.43) |  |
| III | 69(61.06) | 36(63.16) | 33(58.93) |  |
| IV | 22(19.47) | 11(19.30) | 11(19.64) |  |
| Primary prevention, n (%) | 50(44.25) | 32(56.14) | 18(32.14) | 0.0102 |
| Single chamber ICD, n (%) | 53(46.90) | 24(42.11) | 29(51.79) | 0.3025 |
| Dual chamber ICD, n (%) | 16(14.16) | 7(12.28) | 9(16.07) | 0.5633 |
| CRT defibrillator, n (%) | 44(38.94) | 26(45.61) | 18(32.14) | 0.1420 |
| Myocardial infarction, n (%) | 13(11.50) | 6(10.53) | 7(12.50) | 0.7423 |
| Previous CABG or angioplasty, n(%) | 31(27.43) | 14(24.56) | 17(30.36) | 0.4900 |
| Previous valve surgery, n (%) | 6(5.31) | 2(3.51) | 4(7.14) | 0.6586 |
| AF persistent, n (%) | 18(15.93) | 8(14.04) | 10(17.86) | 0.5788 |
| Hypertension, n (%) | 44(38.94) | 20(35.09) | 24(42.86) | 0.3971 |
| Diabetes mellitus, n (%) | 24(21.24) | 11(19.30) | 13(23.21) | 0.6108 |
| Chronic kidney disease, n (%) | 9(7.96) | 5(8.77) | 4(7.14) | 1.0000 |
| COPD, n (%) | 1(0.88) | 0(0.00) | 1(1.79) | 0.4956 |
| LV ejection fraction, % | 36.85(13.10) | 35.38(12.51) | 38.34(13.62) | 0.2309 |
| Medications, n (%) |  |  |  |  |
| β-blocker | 77(68.14) | 41(71.93) | 36(64.29) | 0.3832 |
| ACE inhibitor/ARB | 10(8.85) | 5(8.77) | 5(8.93) | 1.0000 |
| ARNI | 79(69.91) | 40(70.18) | 39(69.64) | 0.9508 |
| Diuretic | 67(59.29) | 37(64.91) | 30(53.57) | 0.2199 |
| Antisterone | 76(67.26) | 42(73.68) | 34(60.71) | 0.1419 |
| Dapagliflozin/Empagliflozin | 50(44.25) | 25(43.86) | 25(44.64) | 0.9332 |
| Class III antiarrhythmic | 23(20.35) | 12(21.05) | 11(19.64) | 0.8524 |
| Anticoagulant | 35(30.97) | 15(26.32) | 20(35.71) | 0.2800 |
| Antiplatelet | 26(23.01) | 15(26.32) | 11(19.64) | 0.3994 |

## **Table S2.** Distribution of clinical interventions among all events for unscheduled visits.

| Clinic/Alert event | Hospitalizations,  n (%) | | Medication changes,  n (%) | | Device programming,  n (%) | | Direct current cardioversion,  n (%) | | Radiofrequency ablation,  n (%) | | Pacing electrode repositioning,  n (%) | |
| --- | --- | --- | --- | --- | --- | --- | --- | --- | --- | --- | --- | --- |
|  | In-Clinic | 5G-CTP | In-Clinic | 5G-CTP | In-Clinic | 5G-CTP | In-Clinic | 5G-CTP | In-Clinic | 5G-CTP | In-Clinic | 5G-CTP |
| Worsening HF or Acute HF | 12 (14.1) | 9 (11.5) | 21 (24.7) | 16 (20.5) | 19 (22.3) | 17 (21.7) | 0 | 0 | 0 | 0 | 0 | 0 |
| Fast V rate during AT/AF: Mean V rate of at least 100 bpm | 0 | 0 | 9 (10.5) | 9 (11.5) | 3 (3.5) | 1 (1.2) | 1 (1.1) | 2 (2.5) | 1 (1.1) | 1 (1.2) | 0 | 0 |
| Shock delivered in an VT/VF episode (at least one) | 0 | 0 | 0 | 4 (5.1) | 13 (15.2) | 15 (19.2) | 0 | 0 | 1 (1.1) | 0 | 0 | 0 |
| Lead impendences out of range | 2 (2.3) | 1 (1.2) | 0 | 0 | 1 (1.1) | 0 | 0 | 0 | 0 | 0 | 1 (1.1) | 1 (1.2) |
| Other events | 0 | 0 | 0 | 0 | 1 (1.1) | 2 (2.5) | 0 | 0 | 0 | 0 | 0 | 0 |

## One event may have two different intervention actions.

## **Table S3.** Distribution of clinical interventions among all events at scheduled follow-up points.

|  | 3-month | | | 6-month | | | 9-month | | | 12-month | | |
| --- | --- | --- | --- | --- | --- | --- | --- | --- | --- | --- | --- | --- |
|  | In-Clinic  (n=102) | 5G-CTP  (n=104) | *P* | In-Clinic  (n=98) | 5G-CTP  (n=103) | *P* | In-Clinic  (n=95) | 5G-CTP  (n=100) | *P* | In-Clinic  (n=94) | 5G-CTP  (n=98) | *P* |
| **All event** | 61(59.80) | 64(61.54) | 0.7989 | 59(60.20) | 67(65.05) | 0.4778 | 34(35.79) | 45(45.00) | 0.1903 | 43(45.74) | 40(40.82) | 0.4908 |
| AT/AF burden at least 6h | 21(20.59) | 20(19.23) | 0.8073 | 19(19.39) | 22(21.36) | 0.7288 | 7(7.37) | 25(25.00) | 0.0009 | 16(17.02) | 16(16.33) | 0.8973 |
| Ventricular rate at least 100 bpm  during AT/AF episodes | 2(1.96) | 3(2.88) | 1.0000 | 1(1.02) | 4(3.88) | 0.3955 | 0(0.00) | 1(1.00) | 1.0000 | 0(0.00) | 1(1.02) | 1.0000 |
| NSVT | 8(7.84) | 8(7.69) | 0.9677 | 10(10.20) | 9(8.74) | 0.7225 | 7(7.37) | 8(8.00) | 0.8686 | 8(8.51) | 5(5.10) | 0.3474 |
| VT/VF detection | 9(8.82) | 13(12.50) | 0.3930 | 0(0.00) | 4(3.88) | 0.1428 | 1(1.05) | 2(2.00) | 1.0000 | 2(2.13) | 3(3.06) | 1.0000 |
| Shock delivered | 1(0.98) | 0(0.00) | 0.4951 | 0(0.00) | 0(0.00) | - | 0(0.00) | 0(0.00) | - | 0(0.00) | 1(1.02) | 1.0000 |
| ATP therapy | 3(2.94) | 4(3.85) | 1.0000 | 0(0.00) | 2(1.94) | 0.4978 | 0(0.00) | 2(2.00) | 0.4978 | 1(1.06) | 3(3.06) | 0.6432 |
| CorVue threshold crossing for lung fluid accumulation | 20(19.61) | 19(18.27) | 0.8063 | 24(24.49) | 30(29.13) | 0.4586 | 16(16.84) | 13(13.00) | 0.4510 | 17(18.09) | 12(12.24) | 0.2586 |
| Lead impedances out of range | 0(0.00) | 0(0.00) | - | 0(0.00) | 0(0.00) | - | 0(0.00) | 0(0.00) | - | 0(0.00) | 0(0.00) | - |
| Sensing problem | 0(0.00) | 0(0.00) | - | 2(2.04) | 3(2.91) | 1.0000 | 1(1.05) | 0(0.00) | 0.4872 | 1(1.06) | 2(2.04) | 1.0000 |
| Threshold problem | 1(0.98) | 1(0.96) | 1.0000 | 5(5.10) | 2(1.94) | 0.4028 | 3(3.16) | 4(4.00) | 1.0000 | 2(2.13) | 2(2.04) | 1.0000 |
| Biventricular pacing ratio below 90% | 6(5.88) | 7(6.73) | 0.8023 | 10(10.20) | 9(8.74) | 0.7225 | 3(3.16) | 2(2.00) | 0.9537 | 5(5.32) | 3(3.06) | 0.6734 |
| **Intervention** |  |  |  |  |  |  |  |  |  |  |  |  |
| Re-programming | 18(29.51) | 20(31.25) | 0.8324 | 12(20.34) | 14(20.90) | 0.9386 | 4(11.76) | 4(8.89) | 0.9658 | 6(13.95) | 7(17.50) | 0.6569 |
| Medicine change | 13(21.31) | 19(29.69) | 0.2835 | 21(35.59) | 20(29.85) | 0.4924 | 10(29.41) | 8(17.78) | 0.2222 | 10(23.26) | 12(30.00) | 0.4867 |
| Hospitalization | 0(0.00) | 1(1.56) | 1.0000 | 1(1.69) | 0(0.00) | 0.4683 | 0(0.00) | 0(0.00) | - | 1(2.33) | 0(0.00) | 1.0000 |
| External direct current cardioversion | 0(0.00) | 0(0.00) | - | 0(0.00) | 0(0.00) | - | 0(0.00) | 0(0.00) | - | 0(0.00) | 0(0.00) | - |
| Radiofrequency ablation | 1(1.64) | 0(0.00) | 0.4880 | 0(0.00) | 0(0.00) | - | 0(0.00) | 0(0.00) | - | 1(2.33) | 0(0.00) | 1.0000 |
| Pacing electrode repositioning | 0(0.00) | 1(1.56) | 1.0000 | 1(1.69) | 0(0.00) | 0.4683 | 0(0.00) | 0(0.00) | - | 0(0.00) | 0(0.00) | - |

## **Table S4.** Etiological Analysis of Medical Resource Utilization in Two Groups.

|  | Group | Acute heart failure | Ventricular rate at least 100 bpm  during AT/AF episodes | Shock delivered in an VT/VF episode | Lead impedances out of range | Other |
| --- | --- | --- | --- | --- | --- | --- |
| Total healthcare uses | In-clinic | 37 | 16 | 14 | 2 | 1 |
|  | 5G-CTP | 35 | 19 | 15 | 1 | 2 |
|  | Total | 72 | 35 | 29 | 3 | 3 |
| Nonurgent in-office visits | In-clinic | 14 | 13 | 0 | 0 | 0 |
|  | 5G-CTP | 18 | 14 | 0 | 0 | 0 |
|  | Total | 32 | 27 | 0 | 0 | 0 |
| Urgent in-office visits | In-clinic | 10 | 1 | 8 | 0 | 1 |
|  | 5G-CTP | 8 | 2 | 9 | 0 | 2 |
|  | Total | 18 | 3 | 17 | 0 | 3 |
| Emergency department visits | In-clinic | 1 | 1 | 5 | 0 | 0 |
|  | 5G-CTP | 0 | 2 | 6 | 0 | 0 |
|  | Total | 1 | 3 | 11 | 0 | 0 |
| Hospitalization | In-clinic | 12 | 1 | 1 | 2 | 0 |
|  | 5G-CTP | 9 | 1 | 0 | 1 | 0 |
|  | Total | 21 | 2 | 1 | 3 | 0 |

This table includes only healthcare interactions related to ICD/CRT-D management (heart failure, arrhythmia, or device-related events)

**Supplementary protocol**

**5G Cloud-Enabled Telemedicine for Remote Management of Heart Failure Patients With Implantable Cardioverter-Defibrillators: A Multicenter Open-Label Randomized Controlled Trial**

**Table of Contents**

[1. Protocol Summary	1](#_Toc23033)

[2. Background	1](#_Toc9362)

[3. Objectives	2](#_Toc24534)

[4. Study endpoints	3](#_Toc12046)

[5. Study Design	5](#_Toc1995)

[6. Study Population	6](#_Toc3605)

[7. Study Interventions and Tools	7](#_Toc24229)

[8. Study Procedures	8](#_Toc17369)

[9. Sample Size Calculation	11](#_Toc19458)

[10. Statistical Analysis Plan	11](#_Toc5870)

[11. Safety Monitoring	12](#_Toc11235)

[12. Ethics and Regulatory Considerations	12](#_Toc27655)

[13. Study Organization	13](#_Toc19974)

[14. References	14](#_Toc8819)

1. **Protocol Summary**

This is a prospective, multicenter, open-label, randomized controlled trial evaluating a 5G-cloud technology platform (5G-CTP) for real-time remote device interrogation and reprogramming versus standard in-clinic care in heart failure (HF) patients with implantable cardioverter-defibrillators (ICDs) or cardiac resynchronization therapy defibrillators (CRT-Ds). A total of 200 patients (100 per group) will be enrolled from five centers across China and randomly assigned (1:1) to 12 months of follow-up. The primary endpoint is the time from clinical event occurrence to clinical intervention initiation. Secondary endpoints include rates of unplanned visits, quality of life (MLHFQ), and healthcare resource utilization.

2. **Background**

Heart failure (HF) imposes a substantial clinical and economic burden,^1^ affecting an estimated 64 million people worldwide, with prevalence increasing because of population aging. Implantable cardioverter-defibrillators (ICDs) and cardiac resynchronization therapy defibrillators (CRT-Ds) are cornerstone therapies for patients with chronic HF who meet guideline-directed indications,^2-4^ significantly reducing mortality and sudden cardiac death. However, the long-term benefits of these cardiac implantable electronic devices (CIEDs) depend on rigorous postoperative follow-up to monitor device function, detect arrhythmic events, and optimize therapeutic parameters.^5^ Current guidelines recommend outpatient visits every 3–6 months,^6,7^ but real-world compliance remains suboptimal, particularly in geographically dispersed populations.

Postoperative follow-up for patients with ICDs/CRT-Ds faces three interrelated challenges. First, ICD/CRT-D programming is highly complex, requiring electrophysiology-trained physicians who can interpret intracardiac electrograms, optimize pacing and defibrillation parameters, and troubleshoot device algorithms. Such expertise is concentrated in urban tertiary centers and remains largely unavailable at community hospitals.^8-10^ Second, rising ICD/CRT-D implantation volumes are straining specialized follow-up clinics, with prolonged appointment wait times and limited time per patient for comprehensive device assessment. Third, delayed detection of critical events remains a major concern: conventional follow-up relies on scheduled every 3–6 months or symptom-driven visits, risking substantial delays between silent but clinically significant events (e.g., lead dysfunction, arrhythmic episodes) and clinical recognition. The TRUST study underscored this inefficiency, revealing that only 6% of scheduled In-clinic visits identified actionable CIED events.^11^ Patients in remote or underserved regions also face geographical and economic barriers, that often reduce follow-up adherence and compromise outcomes.^12-14^

Remote monitoring (RM) has advanced substantially since the 1970s,^15^ from early transtelephonic systems that enabled basic parameter transmission^16,17^ to modern platforms providing automatic data collection and real-time alerts for device integrity issues and arrhythmic events.^18-20^ Randomized trials such as CONNECT and EVOLVO showed that RM reduced time to clinical decision-making and healthcare utilization.^21-23^ However, conventional RM cannot perform remote device reprogramming. When parameter adjustments are required, patients must still undertake in-person clinic visits, a major bottleneck for those residing far from specialized centers. This creates a gap between monitoring and intervention where timely detection is not matched by timely action.

Recent advances in 5G telecommunications and cloud computing offer a practical solution to this monitoring-intervention gap. The 5G-cloud technology platform (5G-CTP) leverages ultra-low latency and high-reliability networks to allow physicians to perform remote device interrogation, testing, and reprogramming in real time. Under this model, patients visit nearby community hospitals where local physicians connect the device to a 5G terminal; remote specialists then access the cloud-based system to perform real-time interrogation, reprogramming, and clinical decision-making. This model eliminates the need for long-distance travel to implantation centers, enables timely intervention for urgent events, and leverages existing community healthcare infrastructure while maintaining specialist-level care.

Despite the promising potential of 5G-CTP, existing evidence is limited to small-scale studies confirming its technical feasibility and safety.^24-26^ Robust randomized controlled trials evaluating whether this platform shortens the time from clinical event detection to intervention remain lacking. Such timeliness represents its core advantage over conventional remote monitoring. This evidence gap is clinically consequential, as prolonged delays in device reprogramming and other therapeutic adjustments for HF patients in remote areas result in avoidable hospitalizations and worsening outcomes. The "Make Changes" study was a prospective, multicenter, randomized controlled trial to evaluate whether 5G-CTP follow-up reduced the time from clinical event occurrence to intervention compared with standard In-clinic care in HF patients with ICDs/CRT-Ds.

3. **Objectives**

We integrated community hospitals with implantation centers via a 5G-cloud technology platform (5G-CTP) to conduct a randomized controlled trial assessing safety, effectiveness, and response time in ICD/CRT-D follow-up.

4. **Study endpoints**

**4.1 Primary endpoints**

The primary endpoint is to evaluate and compare the time from clinical event occurrence to clinical intervention initiation between the 5G-CTP follow-up group and the in-clinic follow-up group. Clinical events are classified into:

- Device-related events: abnormal electrode impedance, CRT% <90%, defibrillator lead impedance issues, prolonged charging times, backup VVI mode activation, pocket/device infections.
- Disease-related events: increased AF burden, ventricular rates >100 bpm during AF, worsening HF, VT/VF events, shock/ATP therapy, CorVue alerts.

Only events requiring clinical intervention are analyzed for the primary endpoint; those without intervention are excluded.

Clinical intervention is defined as modifying pharmacological therapy (e.g., initiating/adjusting HF or anti-arrhythmic drugs), device reprogramming (e.g., increasing pacing output >1V or adjusting algorithms/settings to improve detection of VT/SVT), or planning hospitalizations (e.g., cardioversion or ablation). Detailed flowcharts guide interventions for atrial tachycardia/atrial fibrillation (AT/AF) burden and CorVue Alerts to ensure consistent decision-making.

**4.2 Secondary endpoints**

- To compare proportions of patients requiring emergency or unplanned outpatient visits for HF, arrhythmia, or ICD-related events between groups. The endpoint includes all emergency department visits and unplanned in-office visits, i.e., all visits when the interval between the decision to see the patient and the visit is <24 hours. The events anticipated to prompt these visits were ICD alerts for system integrity, atrial and ventricular arrhythmias, decrease in intrathoracic impedance signifying possible fluid accumulation, and patient symptoms.
- To assess 5G-CTP's impact on quality of life via the MLHFQ at baseline, 6 months, and 12 months.
- To compare healthcare resource utilization, including scheduled visits, unplanned visits, emergency room admissions, and hospitalizations between groups.

Table 1 Minnesota Heart Failure Quality Of Life Scale, MLHFQ

| Item | Scoring | | | | | |
| --- | --- | --- | --- | --- | --- | --- |
|  | No  0 | Very Little  1 | Somewhat  2 | Moderately  3 | A lot  4 | Very much  5 |
| making swelling in your ankles or legs? |  |  |  |  |  |  |
| making you sit or lie down to rest during the day? |  |  |  |  |  |  |
| making your walking about or climbing stairs difficult? |  |  |  |  |  |  |
| making your working around the house or yard difficult? |  |  |  |  |  |  |
| making your going places away from home difficult? |  |  |  |  |  |  |
| making your sleeping well at night difficult? |  |  |  |  |  |  |
| making your relating to or doing things with your friends or family difficult? |  |  |  |  |  |  |
| making your working to earn a living difficult? |  |  |  |  |  |  |
| making your recreational activities or hobbies difficult? |  |  |  |  |  |  |
| making your sexual activities difficult? |  |  |  |  |  |  |
| making you eat less of the foods you like? |  |  |  |  |  |  |
| making you short of breath? |  |  |  |  |  |  |
| making you tired, fatigued, or low in energy? |  |  |  |  |  |  |
| making you stay in the hospital? |  |  |  |  |  |  |
| making you spend money for medical care? |  |  |  |  |  |  |
| giving you side effects from medications? |  |  |  |  |  |  |
| making you feel you are a burden to your family or friends? |  |  |  |  |  |  |
| making you feel a loss of self-control in your life? |  |  |  |  |  |  |
| making you worry? |  |  |  |  |  |  |
| making you feel discouraged or depressed? |  |  |  |  |  |  |
| making you feel a loss of enjoyment of life? |  |  |  |  |  |  |

Scoring Rules: 0 = No, 1 = Very little, 2 = Somewhat, 3 = Moderately, 4 = A lot, 5 = Very much; Total score: sum of all 21 items (range 0–105)

**5. Study Design**

**5.1 Study Type**

The "Make Changes" study is a prospective, multi-center, open-label, randomized controlled trial (RCT; ChiCTR2200059172) in HF patients who have received an implantable ICD or CRT-D. Participants and investigators will not be blinded to group assignment due to the nature of the intervention.

**5.2 Duration of Study**

- Total study duration: 2.5 years, from June 2022 to March 2025.
- Three months for ethical approval and coordination for all centers.
- Patient enrollment at each center: approximately 17 months, from October 2022 to February 2024.
- Follow-up duration: 12 months from enrollment to the last follow-up for all patients.

**5.3 Multicenter Settings**

A total of 5 centers (Changhai Hospital, Shanghai Chest Hospital, Fujian Medical University Union Hospital, Qingdao 971 Hospital, and Xinjiang Military Region General Hospital) nationwide will participate in this study. Operators in each center have considerable experience in surgery for ICD/CRT-D.

**5.4 Randomization and Allocation Concealment**

Patients will be randomly assigned in a 1:1 ratio to either the 5G-CTP group or the In-Clinic group using permuted block randomization with a fixed block size of 4, stratified by enrollment center. The randomization sequence will be generated by an independent statistician prior to study initiation. Allocation concealment will be ensured through the use of sequentially numbered, opaque, sealed envelopes, which will be opened by the investigators responsible for enrolling participants only after confirming the participant's eligibility.

**5.5 Enrollment Modalities**

Enrollment in each subcenter may begin after study initiation and completion of subcenter ethical approval. In this multicenter study, the number of cases enrolled in a single center will not exceed 30% of the total planned number of cases (i.e., 60 cases), and enrollment will be stopped once the upper limit is reached.

**5.6 Study Flow**

**6. Study Population**

All patients with symptomatic HF implanted with Abbott devices (ICD models: CD1377-36C, CD1377-36QC, CD2377-36C, CD2377-36QC; CRT-D models: CD3371-40, CD3371-40Q) who meet all inclusion and have no exclusion criteria are eligible to participate. All enrolled patients are required to sign a written informed consent.

**6.1 Inclusion Criteria**

1. Successful ICD/CRT-D implantation;
2. Residence ≥50 km from the implantation center or follow-up travel time ≥1 hour;
3. Access to a 5G-enabled hospital within 50 km or ≤1 hour travel for follow-up;
4. Able to independently complete the Minnesota Heart Failure Quality of Life Questionnaire (MLHFQ).

**6.2 Exclusion Criteria**

1. Age <18 years;
2. Acute myocardial infarction within the past 90 days;
3. Concurrent or planned enrollment in another trial affecting endpoints;
4. Physician-assessed life expectancy <1 year;
5. Pregnancy or lactation;
6. Cognitive impairments preventing independent questionnaire completion;
7. Inability or unwillingness to provide informed consent.

**7. Study Interventions and Tools**

**7.1 5G-CTP Group**

Patients in the 5G-CTP group will attend outpatient visits at the implantation center at baseline and 12 months, and undergo additional 5G-CTP follow-up at primary hospitals (without local ICD/CRT-D follow-up capability) at months 3, 6, and 9. Through 5G-CTP, the five centers will establish a regional cloud-based follow-up network linking primary hospitals, enabling one-to-one and one-to-many telemonitoring. Primary hospitals will be equipped with a CIED programmer and a 5G remote support terminal.

Previous studies have shown that ICD/CRT-D audio alerts improve clinical event detection, although many detected events are asymptomatic or non-urgent. To reduce anxiety and unnecessary visits, non-critical alerts (excluding lead and device-related warnings) will be disabled.

During 5G-CTP follow-up, patients will visit a primary hospital where a physician places a programmer probe connected to a 5G terminal on the CIED and notifies the remote specialist by phone or SMS. The specialist will access the 5G Cloud Follow-Up APP on a mobile device using two-factor authentication (username/password plus verification code) and remotely interrogate and reprogram the CIED through the 5G platform. If communication fails, the CIED will automatically revert to its baseline settings. The cloud-based system will record session times and programming parameters for audit purposes.

**7.2 In-Clinic Group**

Patients in the In-Clinic group will attend in-person follow-up at the implantation center at baseline and months 3, 6, 9, and 12.

**7.3 Study Tools and Technical Specifications**

The 5G-CTP remote follow-up system is based on the 5G cloud technology support platform, jointly developed with China Telecom. The communication in this system is supported by the fifth generation of mobile communication technology (5G) network offered by China's major telecom carriers. The 5G-CTP can effectively establish a secure connection between the patient's side (programmer, 5G remote support terminal) and the remote side (via mobile phone or tablet installed with a cloud follow-up application, APP), while the whole remote service system is deployed on cloud servers with multilayer protection, including firewalls, customized antivirus, vulnerability scanning, and intrusion detection. To protect data security during transfer, the system utilizes a 2048-bit RSA asymmetric key exchange and autonomous P2P data transfer protocol building on AES encryption protocol.

**8. Study Procedures**

**8.1 Enrollment and Informed Consent**

Enrollment will take place after the screening of the patients or at the same time as the baseline visit. Patients will be enrolled when they meet the inclusion criteria and do not meet the exclusion criteria.

1. Inform eligible patients of study issues and provide written informed consent. The process of obtaining written informed consent must meet the requirements of the Helsinki Declaration, ISO 14155, and local laws and regulations.
2. Written informed consent will be signed and documented by both the patient/authorized person and the investigator.

**8.2 Baseline Visit**

All baseline activities should be performed after the patient has been enrolled and before undergoing surgery for ICD/CRT-D, and should not exceed 30 days after enrollment. The following baseline information will be collected:

1. **Patient demographics and physical findings:** age, gender, follow-up distance, MLHFQ score.
2. **Patient's history of cardiovascular disease:** NYHA class, left ventricular ejection fraction (LVEF).
3. **Patient cardiac medication:** classification of cardiac medications currently taking; documentation of previous antithrombotic medications for atrial fibrillation.
4. **Patient history:** documentation of comorbid heart disease and cardiac surgery; documentation of comorbid non-cardiac conditions.

**8.3 Follow-up Visits**

For 5G-CTP follow-ups, the onsite physician should be able to identify life-threatening changes in ECG, such as severe bradycardia (<40 beats/min), ventricular tachycardia, or ventricular fibrillation. In case of capture loss in a pacemaker-dependent patient, the onsite physician should immediately reprogram the device to emergency VVI pacing by activating the "VVI" key on the programmer. In case of ventricular fibrillation, the onsite physician should immediately activate the "Shock" key on the programmer.

Before the cloud follow-up session, the physician on the patient's side will inform the patient and family of the function, benefits, and risks of cloud follow-up. After obtaining written consent, the local physician and remote specialist will be connected via telephone or video in real-time, while the patient can also communicate with the specialist to eliminate doubts and concerns.

**8.4 Data Collection**

Clinical and device data will be gathered from planned and unplanned visits, whether in-clinic or remote. An event's detection time is set as the moment alert criteria are fulfilled. The resolution or end date of the event is recorded as the date of the physician's clinical action, informed by the device's stored data. This date will be used to accurately calculate the delay from event occurrence to clinical decision-making. If no clinical action follows event confirmation, the event will be excluded from the primary endpoint analysis.

An independent Clinical Event Committee comprising three physicians not involved in the trial will adjudicate all disputed events to ensure accurate classification.

**8.5 Adverse Events**

The definitions of adverse events (AE), adverse device effects (ADE), and serious adverse events (SAE) follow the provisions of ISO 14155:

1. **Adverse event (AE):** any untoward medical occurrence in a patient or clinical study participant.
2. **Adverse Device Effect (ADE):** an adverse event caused by a medical device, including events due to insufficient or inadequate instructions for use or human error.
3. **Serious Adverse Event (SAE):** an adverse event that results in death, life-threatening illness or injury, permanent impairment of body structure or function, hospitalization or prolongation of existing hospitalization, or requires medical or surgical intervention to prevent permanent impairment.

Table 2. Potential Adverse Events and Device-Related Adverse Events

| **Cardiac events** | **non-cardiac events** |
| --- | --- |
| Myocardial infarction | Air embolism |
| pericardial effusion/pericardial tamponade | Reaction to anesthesia |
| Endocarditis | Cerebrovascular accident/TIA |
| Thromboembolism (including device-related) | Infection |
| New-onset or worsening heart failure | Vasovagal reaction |
| Hypotensive shock | Pneumothorax |
| Vascular damage/insufficiency | pulmonary edema |
| Ventricular arrhythmias requiring defibrillation | Pulmonary embolism |
| Pacemaker/ICD components damage | Pleural effusion |
| Atrial tachyarrhythmias requiring intervention | Respiratory distress |
|  | Syncope |

**Recording and Reporting Processes:**

1. Safety monitoring begins from enrollment, whether randomized or not.
2. All SAEs and serious ADEs will be reported to the study leader immediately upon occurrence.
3. Adverse event information will be recorded in the hospitalization record and eCRF as soon as possible.
4. Adverse events must be reported to the Ethics Committee (EC) according to local regulations.

**8.6 Protocol Deviations**

Protocol deviation is defined as a failure to follow the study protocol, including:

1. Patient Informed Consent (PIC) not approved by the Ethics Committee;
2. Patient did not sign PIC or date not indicated;
3. Study-specific procedure occurred before patient signed PIC;
4. Required study visit not conducted.

In case of protocol deviation, the deviation will be recorded in the CRF deviation table, and the study leader will be notified.

**9. Sample Size Calculation**

Previous research shows the 5G-CTP follow-up group had a mean time of 6 days (SD = 4.9 days), while the in-clinic follow-up group had a mean of 22 days (SD = 29.9 days). With a bilateral α of 0.05 and 90% power, initial calculations suggested at least 40 participants per group. Considering an approximate 50% event rate during follow-up, the number was increased to 80 per group. With an additional 20% reserve for anticipated dropout, the final target is 100 subjects per group (total n = 200).

**10. Statistical Analysis Plan**

All analyses will be conducted using the Intent-to-Treat (ITT) population. Continuous variables will be summarized using means and standard deviations (SD) for normally distributed data, or medians and interquartile ranges (IQR) for non-normal data. Categorical variables will be reported as frequencies and percentages. Comparisons between groups will be made using the chi-squared test, t-test, or Wilcoxon rank-sum test, depending on data distribution and type.

For the primary outcome (time from clinical event to intervention), mixed-effects models will estimate the least squares mean difference and 95% confidence interval (CI) between groups, treating patients and centers as random effects. The same approach will be applied to compare programming times. Given repeated MLHFQ measurements, a mixed-effects model with a group-by-time interaction term will assess trends in index changes and estimate the least squares mean difference and 95% CI at 12 months. No imputation will be performed for missing data due to loss to follow-up.

Subgroup analyses for the primary outcome will be conducted based on center, sex, age (<60 vs. ≥60 years), NYHA classification, ischemic cardiomyopathy, baseline LVEF (≤35% vs. >35%), persistent AF, pacemaker type, and primary ICD prevention.

Statistical analyses will be performed using R (version 4.0.3) and SAS 9.4 (SAS Institute Inc., Cary, NC). A two-tailed P-value < 0.05 will be considered statistically significant.

**11. Safety Monitoring**

To ensure effective patient safety protection:

- A dual password authentication login is utilized. The remote specialist logs into the designated account and uses an access password for second verification to log into the cloud follow-up APP.
- The whole remote service system is deployed on cloud servers with multilayer protection (firewalls, customized antivirus, vulnerability scanning, intrusion detection).
- Data transfer security is ensured via 2048-bit RSA asymmetric key exchange and autonomous P2P data transfer protocol based on AES encryption.
- Trained personnel will be available for emergency support to ensure network reliability and on-site safety.

**12. Ethics and Regulatory Considerations**

**12.1 Ethics Approval**

This study will follow the Declaration of Helsinki, ISO 14155, and all local laws and regulations. Before the start of the study, the clinical study plan will be submitted to the relevant Ethics Committee (EC)/Institutional Review Board (IRB) for review together with relevant documents (patient information sheet, informed consent form). EC/IRB approval documents must include:

- Date of the meeting;
- Composition of the Committee and list of participants and voters;
- Clinical study protocol version reviewed;
- Patient information and informed consent version reviewed.

**12.2 Informed Consent**

Written informed consent will be obtained from all participants before enrollment. The consent process will comply with the Helsinki Declaration and ISO 14155.

**12.3 Data Privacy and Security**

All patient data will be handled in accordance with applicable data protection laws. Cloud servers feature multilayer security including firewalls, antivirus, and intrusion detection.

**12.4 Data Sharing Plan**

One year following the publication of the study, de-identified trial data will be made available upon request to the corresponding author. Requesters are required to clearly outline the specific purpose for data use. A signed data access agreement will be mandatory.

**12.5 Publication Policy**

At the end of the study, a clinical and statistical report will be prepared by the study leader in consultation with co-investigators. The first publication should cover the full range of data from the study centers. Publication of any part of the findings by or on behalf of the investigator should be approved by the Head of Study prior to submission. The first published article will be written by the sponsor center as the corresponding author and by the top 3 centers with the highest number of enrolled cases as co-first authors. All authors will be listed in descending order of enrollment contribution.

**13. Study Organization**

**13.1 Study Leadership**

Professor Zhongkai Wang of the First Affiliated Hospital of Naval Medical University, Shanghai, China, is responsible for initiating, executing, and coordinating the study. Abbott coordinates and helps with study enrollment and follow-up, including recognition of clinical investigators, activation of study centers, development and analysis of study databases, and review of collected data.

**13.2 Investigator Responsibilities**

Investigators will:

- Provide written authorization;
- Use EC-approved informed consent;
- Follow the study plan, ISO 14155, and local regulations;
- Collect and store postoperative and follow-up data;
- Screen and enroll suitable patients;
- Support monitors and reviewers to ensure strict implementation of the clinical investigation plan (CIP).

**13.3 Monitoring**

As the initiator, Changhai Hospital will ensure strong supervision of the study process to ensure smooth implementation, recording, and reporting according to the CIP and relevant laws. Supervision will be carried out according to standard operating procedures.

**14. References**

1. Dickstein K, Vardas PE, Auricchio A, et al. 2010 Focused Update of ESC Guidelines on device therapy in heart failure. *Eur Heart J*. 2010;31(21):2677-87.
2. Bardy GH, Lee KL, Mark DB, et al. Amiodarone or an implantable cardioverter-defibrillator for congestive heart failure. *N Engl J Med*. 2005;352(3):225-37.
3. Cleland JGF, Daubert JC, Erdmann E, et al. The effect of cardiac resynchronization on morbidity and mortality in heart failure. *N Engl J Med*. 2005;352(15):1539-49.
4. Epstein AE, DiMarco JP, Ellenbogen KA, et al. ACC/AHA/HRS 2008 Guidelines for Device-Based Therapy of Cardiac Rhythm Abnormalities. *Circulation*. 2008;117(21):e350-408.
5. Kirkfeldt RE, Johansen JB, Nohr EA, et al. Complications after cardiac implantable electronic device implantations. *Eur Heart J*. 2014;35(18):1186-94.
6. Carlson MD, Wilkoff BL, Maisel WH, et al. Recommendations from the Heart Rhythm Society Task Force on Device Performance Policies and Guidelines. *Heart Rhythm*. 2006;3(10):1250-73.
7. Slotwiner D, Varma N, Akar JG, et al. HRS Expert Consensus Statement on remote interrogation and monitoring for cardiovascular implantable electronic devices. *Heart Rhythm*. 2015;12(7):e69-100.
8. Ferrick AM, Raj SR, Deneke T, et al. 2023 HRS/EHRA/APHRS/LAHRS expert consensus statement on practical management of the remote device clinic. *Heart Rhythm*. 2023;20(9):e92-e144.
9. Winters SL, Packer DL, Marchlinski FE, et al. Consensus statement on indication, guidelines for use, and recommendations for follow-up of implantable cardioverter defibrillators. *Pacing Clin Electrophysiol*. 2001;24(2):262-269.
10. Wilkoff BL, Auricchio A, Brugada J, et al. HRS/EHRA expert consensus on the monitoring of cardiovascular implantable electronic devices. *Heart Rhythm*. 2008;5(6):907-925.
11. Varma N, Epstein AE, Irimpen A, et al. Efficacy and safety of automatic remote monitoring for implantable cardioverter-defibrillator follow-up: the TRUST trial. *Circulation*. 2010;122(4):325-32.
12. Haugaa KH, Dan GA, Iliodromitis K, et al. Management of patients with ventricular arrhythmias and prevention of sudden cardiac death. *Europace*. 2018;20(FI2):f249-f253.
13. Poole JE, Olshansky B, Mark DB, et al. Long-Term Outcomes of Implantable Cardioverter-Defibrillator Therapy in the SCD-HeFT. *J Am Coll Cardiol*. 2020;76(4):405-415.
14. Sapp JL, Sivakumaran S, Redpath CJ, et al. Long-Term Outcomes of Resynchronization-Defibrillation for Heart Failure. *N Engl J Med*. 2024;390(3):212-220.
15. Furman S, Parker B, Escher DJ. Trans-telephone pacemaker clinic. *J Thorac Cardiovasc Surg*. 1971;61(5):827-34.
16. Federico AJ, Giori F, Bhayana JN, et al. Instrumentation for the follow-up of pacemaker patients. *Pacing Clin Electrophysiol*. 1979;2(3):315-24.
17. Dreifus LS, Zinberg A, Hurzeler P, et al. Transtelephonic monitoring of 25,919 implanted pacemakers. *Pacing Clin Electrophysiol*. 1986;9(3):371-8.
18. Lazarus A. Remote, wireless, ambulatory monitoring of implantable pacemakers, cardioverter defibrillators, and cardiac resynchronization therapy systems. *Pacing Clin Electrophysiol*. 2007;30 Suppl 1:S2-S12.
19. Lunati M, Gasparini M, Santini M, et al. Follow-up of CRT-ICD: implications for the use of remote follow-up systems. *Pacing Clin Electrophysiol*. 2008;31(1):38-46.
20. Marzegalli M, Lunati M, Landolina M, et al. Remote monitoring of CRT-ICD: the multicenter Italian CareLink evaluation. *Pacing Clin Electrophysiol*. 2008;31(10):1259-64.
21. Landolina M, Perego GB, Lunati M, et al. Remote monitoring reduces healthcare use and improves quality of care in heart failure patients with implantable defibrillators: the EVOLVO study. *Circulation*. 2012;125(24):2985-92.
22. Perl S, Stiegler P, Rotman B, et al. Socio-economic effects and cost saving potential of remote patient monitoring (SAVE-HM trial). *Int J Cardiol*. 2013;169(6):402-7.
23. Varma N, Marrouche NF, Aguinaga L, et al. HRS/EHRA/APHRS/LAHRS/ACC/AHA Worldwide Practice Update for Telehealth and Arrhythmia Monitoring. *Circ Arrhythm Electrophysiol*. 2020;13(7):e009007.
24. Xiong S, Li J, Tong L, et al. Realtime Remote Programming in Patients Carrying Cardiac Implantable Electronic Devices Requiring Emergent Reprogramming. *Front Cardiovasc Med*. 2022;9:871425.
25. Tong L, Long Y, Xiong S, et al. Application of Postoperative Remote Follow-Up of CIED Based on the 5G Cloud Technology Support Platform in Areas With Underdeveloped Medical Resources. *Front Cardiovasc Med*. 2022;9:894345.
26. Tong L, Xiong S, Hou J, et al. Cloud Follow-Up in Patients With Cardiovascular Implantable Electronic Devices: A Single-Region Study in China. *Front Cardiovasc Med*. 2022;9:864398.
27. Hu SS. Disorders of cardiac rhythm in China. *J Geriatr Cardiol*. 2024;21(7):703-712.
28. Cleland J, Freemantle N, Ghio S, et al. Predicting the long-term effects of cardiac resynchronization therapy on mortality from baseline variables and the early response: the CARE-HF Trial. *J Am Coll Cardiol*. 2008;52(6):438-45.
29. Boriani G, Da Costa A, Ricci RP, et al. The MORE-CARE randomized controlled trial: phase 1 results on dynamics of early intervention with remote monitoring. *J Med Internet Res*. 2013;15(8):e167.
30. Crossley GH, Boyle A, Vitense H, et al. The CONNECT trial: the value of wireless remote monitoring with automatic clinician alerts. *J Am Coll Cardiol*. 2011;57(10):1181-9.
31. Rumsfeld JS, Alexander KP, Goff DC Jr, et al. Cardiovascular health: the importance of measuring patient-reported health status. *Circulation*. 2013;127(22):2233-49.
32. Anker SD, Agewall S, Borggrefe M, et al. The importance of patient-reported outcomes: a call for their comprehensive integration in cardiovascular clinical trials. *Eur Heart J*. 2014;35(30):2001-9.
33. Guédon-Moreau L, Lacroix D, Sadoul N, et al. A randomized study of remote follow-up of implantable cardioverter defibrillators: safety and efficacy report of the ECOST trial. *Eur Heart J*. 2013;34(8):605-14.
34. Parthiban N, Esterman A, Mahajan R, et al. Remote Monitoring of Implantable Cardioverter-Defibrillators: A Systematic Review and Meta-Analysis of Clinical Outcomes. *J Am Coll Cardiol*. 2015;65(24):2591-2600.
35. Veldhuisen DJ van, Braunschweig F, Conraads V, et al. Intrathoracic impedance monitoring, audible patient alerts, and outcome in patients with heart failure. *Circulation*. 2011;124(16):1719-26.
36. Boriani G, Auricchio A, Klersy C, et al. Healthcare personnel resource burden related to in-clinic follow-up of cardiovascular implantable electronic devices. *Europace*. 2011;13(8):1166-1173.
37. Lazarus A. Remote, wireless, ambulatory monitoring of implantable pacemakers, cardioverter defibrillators, and cardiac resynchronization therapy systems: analysis of a worldwide database. *Pacing Clin Electrophysiol*. 2007;30 Suppl 1:S2-S12.
